# miR-24 Regulates Intrinsic Apoptosis Pathway in Mouse Cardiomyocytes

**DOI:** 10.1371/journal.pone.0085389

**Published:** 2014-01-15

**Authors:** Li Wang, Li Qian

**Affiliations:** 1 Department of Pathology and Laboratory Medicine; University of North Carolina, Chapel Hill, North Carolina, United States of America; 2 McAllister Heart Institute, University of North Carolina, Chapel Hill, North Carolina, United States of America; Mayo Clinic, United States of America

## Abstract

Numerous cardiac diseases, including myocardial infarction (MI) and chronic heart failure, have been associated with cardiomyocyte apoptosis. Promoting cell survival by inhibiting apoptosis is one of the effective strategies to attenuate cardiac dysfunction caused by cardiomyocyte loss. miR-24 has been shown as an anti-apoptotic microRNA in various animal models. *In vivo* delivery of miR-24 into a mouse MI model suppressed cardiac cell death, attenuated infarct size, and rescued cardiac dysfunction. However, the molecular pathway by which miR-24 inhibits cardiomyocyte apoptosis is not known. Here we found that miR-24 negatively regulates mouse primary cadiomyocyte cell death through functioning in the intrinsic apoptotic pathways. In ER-mediated intrinsic pathway, miR-24 genetically interacts with the CEBP homologous gene CHOP as knocking down of CHOP partially attenuated the induced apoptosis by miR-24 inhibition. In mitochondria–involved intrinsic pathway, miR-24 inhibits the initiation of apoptosis through suppression of Cytochrome C release and Bax translocation from cytosol to mitochondria. These results provide mechanistic insights into the miR-24 mediated anti-apoptotic effects in murine cardiomyocytes.

## Introduction

Cardiac disease is the leading cause of death and disability in the developed countries. In the US over five million patients suffer from progressive cardiac dysfunction, known as heart failure. A variety of animal and human studies have demonstrated that apoptosis (programmed cell death) contributes significantly to cardiomyocyte loss during the development and progression of heart failure [1], [2]. Because cardiomyocytes are terminally differentiated and have little potential for division, preventing cell death has important implications in the treatment of cardiovascular disease [3]. Introduction of new cardiac cells by cell-based therapeutic approaches is promising, but promoting survival of newly introduced cardiomyocytes remains challenging [4], [5], [6].

Apoptosis is a highly conserved and regulated cell death process that plays a fundamental role in myriad physiological processes. The diverse stresses and conditions that trigger apoptosis ultimately converge to activate a family of aspartic acid–specific cysteine proteases, called caspases [7], [8]. Activation of caspases is central to apoptosis and can be initiated by any of three distinct mechanisms: (1) ligand binding to death receptors, (2) release of Cytochrome C from mitochondria, and (3) stress to the endoplasmic reticulum (ER) [9], [10], [11].

Alternatively, apoptosis can be triggered through either the extrinsic pathway or the intrinsic pathway. The extrinsic pathway is initiated through the stimulation of the transmembrane death receptors, such as the Fas receptors, located on the cell membrane. In contrast, the intrinsic pathway is initiated from within the cell by developmental cues or severe cell stress [7], [8]. The mitochondria is considered the central organelle in the intrinsic apoptotic pathway[10]. However, accumulating evidence suggests that other organelles, such as the ER, lysosomes, and Golgi apparatus, are also involved in bridging the pro-apoptotic signaling with cellular stress [9], [12]. In the myocardium, the ER participates in stress-induced apoptosis, cellular calcium homeostasis, and synthesis of secretory proteins, such as atrial natriuretic peptide, brain natriuretic peptide, and vascular endothelial growth factor [13], [14], [15]. Dysfunction of the ER might thus contribute to the pathogenesis of heart disease [13].

Members of the Bcl-2 family proteins are major regulators of the intrinsic apoptotic pathway and play an important role in regulating cardiomyocyte apoptosis [16]. The family includes pro-apoptotic (e.g., Bax, Bid, Bim) and anti-apoptotic (e.g., Bcl-2, Bcl-xL) members [16], [17]. Overexpression of anti-apoptotic Bcl-2 family proteins protects cardiomyocytes from doxorubicin and hypoxia-induced cell death [16]. Bax, the Bcl2-associated X protein, forms heterodimers with Bcl-2 and promotes programmed cell death [18], [19]. Bax is generally sequestered in the cytosol and trafficked from the cytosol into mitochondria upon apoptotic stimuli [20], [21], [22]. Bax translocation into mitochondria triggers the release of cytochrome c [23], [24]. The released cytochrome c from the mitochondria cleaves and activates caspase-3 and caspase-9 to trigger a series of downstream apoptotic events [21], [22], [24].

A more recently discovered mechanism of post-transcriptional regulation involves a class of small non-coding RNAs, known as microRNAs (miRNAs) (for reviews [25], [26], [27], [28]). By imperfect sequence-specific binding to their mRNA targets, miRNAs negatively regulates protein expression by degrading target mRNA or inhibiting translation. miR-24 is one of the microRNAs that functions in multiple biological processes, including erythroid differentiation, DNA-repair process, cell cycle regulation and programmed cell death [29], [30], [31], [32], [33], [34]. Especially, we and others showed that miR-24 negatively regulates apoptosis in frogs and mice [33], [35]. Here we studied the molecular mechanisms by which miR-24 inhibits apoptosis in murine primary cardiomyocytes. By performing a series of epistasis analyses, we found that miR-24 modulated intrinsic apoptotic pathway including both ER and mitochondria-involved apoptosis.

## Materials and Methods

### Primary cardiomyocyte culture and transfection

Primary cardiomyocytes from mouse neonatal hearts were isolated and maintained as described [36]. Animal protocol was approved by UNC-Chapel Hill DLAM. All procedures conform to NIH guidelines. Briefly, hearts were minced and digested with collagenase type II (Worthington) solution. Digested cells were pre-plated for 2 hr to enrich cardiomyocytes. The attached cells after 2hr plating were considered to be non-myocytes and discarded, while the unattached cells were primarily cardiomyocytes. The cardiomyocyte identity was further confirmed by immunocytochemistry with myocardial markers. Unattached cells were cultured in DMEM/M199 medium containing 10% FBS at a density of 10^4^/cm^2^. Lipofectamine 2000 (Invitrogen)-mediated transfection was performed according to Invitrogen's protocol. miR-24 mimic (5′-UGGCUCAGUUCAGCAGGAACAG-3′), mimic control (5′-UUCUCCGAACGUGUCACGUTT-3′), miR24^mut^ mimic (5′-UGGCUCAGUUCAGUAAGAACCG-3′), miR-24 inhibitor (5′-ACCGAGUCAAGUCGUCCUUGUC-3′), inhibitor control (5′-UCUACUCUUUCUAGGAGGUUGUGA-3′) and miR24^mut^ inhibitor (5′-ACCGAGUCAAGUCAUUCUUGGC-3′) were purchased from Dharmacon and Shanghai GenePharma Co. siRNA cocktails against Caspase3, Caspase8, Caspase9, Caspase12, Bim, ATF6, CHOP, JNK, XBP1 were purchased from Dharmacon (ThermoScientific) as SmartPool OnTarget siRNAs. For transfection in each well of a six-well plate, 40 pmol of oligos was used.

### Chemical reagents

Tunicamycin and thapsigargin were purchased from Sigma and resuspended in DMSO. Tunicamycin was used at 0.1 µg/ml, and thapsigargin was used 0.1 µM, according to previous studies [15], [37]. CD95 and camptothecin were purchased from BD Pharmingen and used at 5 µM per manufacturer's protocol. Caspase Inhibitor VI (Z-VAD-FMK) that inhibits all caspases was purchased from Calbiochem. Caspase-8/FLICE inhibitor (Z-IETD-FMK), Caspase-9/Mch6 inhibitor (Z-LEHD-FMK) and Caspase-12 inhibitor (Z-ATAD-FMK) were purchased from Biovision. All caspase inhibitors were dissolved in DMSO and kept at 2 mM as 1000X stock solution. For cell treatments, the aforementioned chemicals were diluted directly in corresponding media and filtered for sterile conditions.

### Flow cytometry

To detect early apoptotic cells (AnnexinV+PI-), dissociated cells (5×10^5^) were washed twice in PBS and resuspended in 1Xbinding buffer (BD Biosciences). The cells were then stained with AnnexinV-FITC and propidium iodide (PI) (ready-to-use solutions, BD Biosciences) for 30 minutes in dark and followed by FACS analysis using Calibur (BD Biosciences).

### Quantitative real-time PCR

Total RNA was extracted with the TRizol method (Invitrogen). RT-PCR was performed using Superscript III first-strand synthesis system (Invitrogen). qPCR was performed using the ABI 7900HT (TaqMan, Applied Biosystems) per the manufacturer's protocols. Optimized primers from Taqman Gene Expression Array were used. MicroRNA RT was conducted using Taqman MicroRNA Reverse Transcription Kit (Applied biosystems). MicroRNA real time PCR (qRT-PCR) was performed per the manufacturer's protocols by using primers from Taqman MicroRNA Assays (Applied biosystems). Expression levels were normalized to Gapdh expression and RNU6 (microRNA qPCR).

### Semi-quantitative RT-PCR

XBP1 mRNA splicing was determined using semi-quantitative RT-PCR. The established primers to detect the unspliced form and spliced form are used as followed: XBP1 forward, 5′- CAG ACT ACG TGC GCC TCT GC -3′; XBP1 reverse 5′- CTT CTG GGT AGA CCT CTG GG -3′; sXBP1 forward 5′- TCT GCT GAG TCC GCA GCA GG -3′; sXBP1 reverse 5′- CTC TAA GAC TAG AGG CTT GG -3′. GAPDH was used as an endogenous control, the primers are: forward, 5′- CAT CAA CGA CCC CTT CAT TGA CCT CAA CTA -3′; reverse, 5′- TCC ACG ATG CCA AAG TTG TCA TGG ATG ACC -3′.

### Western blot

Western blots were performed as described [38]. Mouse monoclonal anti-Caspase 8 (Sigma), mouse monoclonal anti-Caspase 9 (Sigma), rabbit anti-Caspase 3 (Sigma), rat monoclonal anti-Caspase 12 (Sigma), rabbit polyclonal antibody against Bim (amino acids 4–195 of Bim_EL_ form), rabbit anti-Cytochrome C (Cell Signaling), goat polyconal anti-HP1 (Santa Cruz), rabbit anti-CoxIV (Cell Signaling), rabbit anti-phospho-JNK (Thr183/Tyr185) (81E11) (Cell Signaling), rabbit anti-JNK (Cell Signaling), mouse monoclonal anti-CHOP (L63F7) (Cell Signaling), rabbit anti-Apaf-1 (Cell Signaling), rabbit anti-Bcl2 (Cell Signaling), rabbit polyclonal anti-ATF-6 (Santa Cruz), rabbit anti-Bax (Cell Signaling) were all used at a 1:1000 dilution for western blots.

## Results

### miR-24 functions in the intrinsic apoptosis pathway

**Figure 1:**
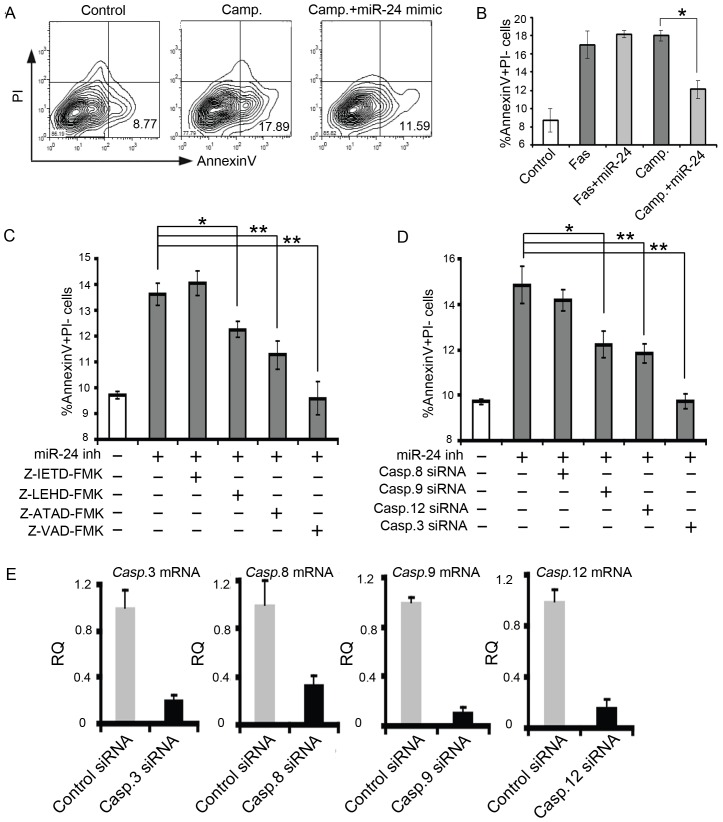
miR-24 inhibits the intrinsic apoptosis pathway. (A) FACS analysis on AnnexinV^+^PI^–^ - apoptotic cells treated with Camp with (middle panel) or without miR-24 mimic (right panel). The control group is shown in the left panel. Camp., Camptothecin (B) Quantification on AnnexinV^+^PI^–^ cells treated with Fas or Camp with or without miR-24 mimic. (C) Inhibition of Caspase 9, Caspase 12 or Caspase 3, but not caspase 8, rescued the miR-24 loss-of-function phenotype. Cells were incubated with Z-IETD-FMK, Z-LEHD-FMK, Z-ATAD-FMK or Z-VAD-FMK for assessment of caspase-8, -9, -12 and pan caspases activity, respectively. (D) Knockdown of *Caspase 9, Caspase 12* or *Caspase 3*, but not *Caspase 8*, rescued the increased apoptosis caused by inhibition of miR-24. *, p<0.05; **, p<0.01. Data were analyzed by unpaired Student's t test. (E) Validation of knockdown efficiency using siRNA cocktail against *Caspase 3, 8, 9* and *12* by qPCR. RQ, Relative Quantification. Error bars represent SEM.

We and others have previously shown that miR-24 negatively regulates apoptosis in several different cell types including cardiomyocytes both *in vitro* and *in vivo*. We demonstrated that miR-24 level is acutely down-regulated upon cardiac injury, *in vivo* delivery of miR-24 confers protective effects in infarcted heart [35]. However the molecular pathway involving miR-24 is largely unknown. Given the critical anti-apoptotic role of miR-24 in the ischemic heart, it is important to determine the mechanisms by which miR-24 inhibits apoptosis in cardiomyocytes. Apoptosis can be induced via the intrinsic pathway, which involves Bcl-2 family proteins, or via the extrinsic pathway, which involves death receptors such as CD95 (Fas) [7], [8]. To determine in which apoptotic pathway miR-24 functions, we treated the cardiomyocytes with CD95/Fas or Campothecin to induce apoptosis by activating the extrinsic or intrinsic pathway, respectively. Subsequently, we delivered miR-24 mimic using our established protocol and optimized dosage [35]. The induction of miR-24 level was confirmed and the specificity of the mimic/inhibitor was validated using luciferase sensor experiment as documented before [35]. The treated cells were then stained with AnnexinV and propidium iodide (PI) followed by flow cytometric analysis to determine the rate of apoptosis. We found that introduction of miR-24 significantly attenuated the increased percentage of AnnexinV+PI- early apoptotic cells induced by camptothecin (18% to 12%, p<0.05), but not by Fas (18% to 17%, p>0.05) (Fig. 1A and B). These data suggest that expression of miR-24 inhibited camptothecin-induced intrinsic apoptosis but not Fas-induced extrinsic apoptosis.

The extrinsic pathway via the death receptor involves Caspase 8, while the intrinsic mitochondrial pathway activates Caspase 9, and endoplasmic reticulum (ER) stress-mediated apoptosis activates Caspase12 [7], [8]. To confirm the observation we made using Fas and Camptothecin and further dissect the apoptotic pathways involving miR-24, we treated the miR-24 inhibitor (tested and validated in [35]) transfected cardiomyocytes with a series of Caspase inhibitors and assessed the degree of apoptosis inhibition. Consistent with the Fas data, inhibition of Caspase 8 using Z-IETD-FMK did not alleviate the increased apoptosis induced by miR-24 inhibition. Interestingly, inhibiting Caspase 9 or Caspase 12, both of which are involved in the intrinsic apoptosis, significantly decreased the percentage of apoptotic cells induced by miR-24 inhibition (Fig.1C). Inhibiting all Caspases by Z-VAD-FMK completely rescued the apoptotic effects caused by miR-24 inhibition. As an alternative method, we utilized the siRNA SmartPool (see method, a siRNA cocktail containing 3∼5 different siRNA sequences against one gene to minimize the off-target effect) to knock-down the Caspase genes individually to examine the effect on induced apoptosis by miR-24 inhibition. Knocking-down efficiency of each Caspase was validated by qPCR. We observed consistent 70-90% reduction in the expression of each gene with the siRNA SmartPool (Fig.1E). Knocking-down Caspase 9 or Caspase 12, but not Caspase 8, resulted in a partial rescue of increased apoptosis caused by miR-24 inhibitor. Meanwhile, knocking-down the final “executor Caspase” -Caspase 3 completely reversed the increased apoptosis by miR-24 inhibition (Fig. 1D). Therefore, these data collectively suggest that miR-24 regulates intrinsic but not extrinsic apoptosis.

### miR-24 modulates ER-mediated intrinsic apoptosis

**Figure 2:**
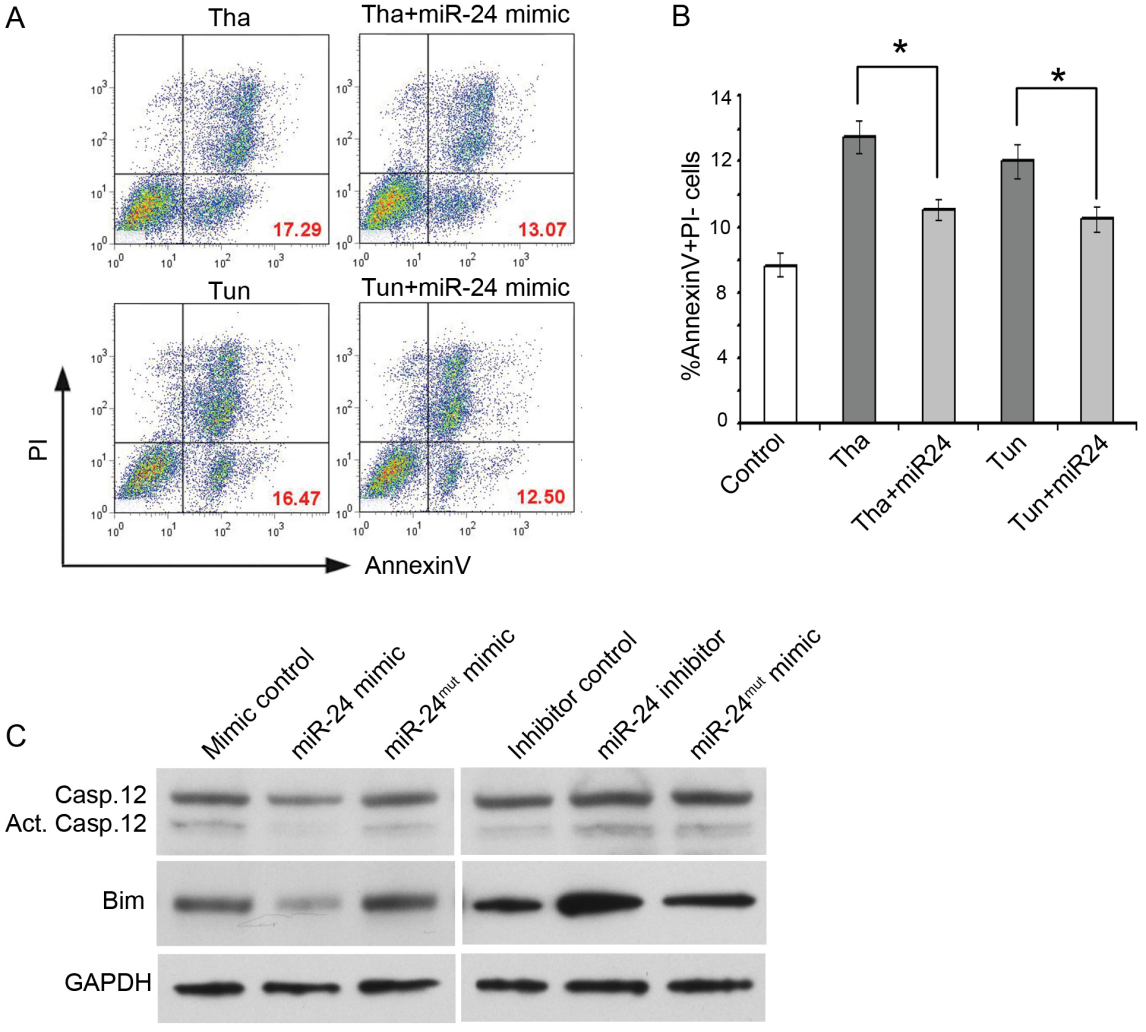
miR-24 modulates ER-mediated apoptosis pathway. (A) miR-24 attenuates thapsigargin and tunicamycin induced cell death through ER-mediated apoptosis pathway. Mouse cardiomyocytes with or without overexpression of miR-24 were cultured with thapsigargin or tunicamycin for six hours. Cells were stained with FITC-conjugated Annexin V and PI for flow cytometry. Tha, thapsigargin; Tun, tunicamycin. (B) Percentage of AnnexinV^+^PI^–^ cells in Fig. 2A shown as mean ± SEM. These data are representative of three independent experiments. *, p<0.05. Data were analyzed by unpaired Student's t test. (C) Western blot for Caspase 12 and Bim from primary CMs transfected with miR-24 mimic, inhibitor, or corresponding controls. GAPDH serves as a loading control.

In general, intrinsic apoptotic pathways can be initiated in ER or mitochondria of the cardiomyocytes. Next, we wanted to investigate the contribution of miR-24 to distinct intrinsic apoptotic pathways. To induce ER stress-mediated apoptosis, we challenged the cardiomyocytes pharmacologically with tunicamycin and thapsigargin. Tunicamycin is an inhibitor of N-glycosylation in the ER, while thapsigargin disrupts intracellular calcium homeostasis. As previously reported, treatment of cardiomyocytes with tunicamycin or thapsigargin caused an increase in the percentage of AnnexinV+ cells and Caspase-12 expression and cleavage (Fig.2 and data not shown) [39]. To test whether miR-24 could attenuate ER stress-induced apoptosis, we expressed miR-24 in cardiomyocytes treated with tunicamycin or thapsigargin. Compared to mock transfection, miR-24 expression significantly inhibited tunicamycin or thapsigargin induced apoptosis (Fig.2A and B).

It is generally considered that Caspase-12 is the initiator caspase in ER-stress-mediated apoptosis based on the observation that Caspase-12 processing (synthesized and cleaved) occurs during ER-stress induced apoptosis. Therefore, we tested whether miR-24 had impact on the Caspase-12 expression level and processing. By performing western blot, we found protein levels of both non-cleaved and cleaved forms of Caspase 12 were reduced when miR-24 was overexpressed (Fig. 2C). In contrary, accumulation of both forms of Caspase 12 was observed when miR-24 inhibitor was introduced into the cells.

MicroRNAs regulate downstream targets by imperfect binding of their “seed sequence”, the 6-8 nucleotides at the 5′ end of a miRNA that is thought to be an important determinant of target specificity, to the 3′ UTR of target genes. To determine that the regulation of miR-24 on Caspase expression is indeed through binding of its seed sequence to downstream targets, we designed additional controls where the seed sequence of miR-24 mimic and inhibitor were mutated. We rationalize that if the alteration in protein levels is indeed due to the changes of miR-24 activity, the effects we observed using miR-24 mimic or inhibitor would be abolished when using mutated forms of mimic or inhibitor. Indeed, when we transfected cells with miR-24^mut^ mimic and inhibitor, the alteration in proteins levels of Caspase12 and Bim with miR-24 mimic or inhibitor was diminished suggesting the effects caused by miR-24 mimic and inhibitor are specific (Fig.2C). Furthermore, we performed luciferase assay to test if miR-24 directly regulates Caspase 12 through binding to its 3′UTR. Co-transfection of miR-24 mimic or inhibitor with the luciferase reporter that contains the 3′UTR region of Caspase 12 did not result in a significant change in the luciferase activity when compared to the controls (Figure S1). These data suggest that the inhibition of Caspase 12 protein levels by miR-24 is indirect, possibly through Bim or other direct target(s) in this pathway.

### Anti-apoptotic effect of miR-24 overexpression is associated with decreased CHOP activity in the ER pathway

Based on the data above, we conclude that miR-24 is involved in regulating ER-mediated apoptosis in cardiomyocytes. Next, we want to identify the specific pathway(s) in ER-mediated apoptosis that involves miR-24. One of the downstream effects of ER stress is the activation of unfolded protein response (UPR). The accumulation of improperly folded proteins in the ER leads to adaptive responses, collectively known as UPR that induces the expression of genes encoding protein chaperones and folding catalysts. The ER stress-induced transcription factors activating transcription factor 6 (ATF6) and X-box binding protein 1 (XBP1) serve to up-regulate ER chaperone proteins and acts upstream to other UPR pathways. In order to determine whether miR-24 exerts its effects on ER pathway by regulating UPR, we examined several key events in UPR in the presence and absence of miR-24. First, we determined if altered expression of miR-24 affected the transcription and translation of ATF6 by qPCR and western blot. We found overexpression or inhibition of miR-24 did not affect ATF6 mRNA or protein level (Figure S2A). Next we determined if miR-24 modulates the translocation of ATF6 from the cytosol to nucleus, as the nuclear localization of ATF6 is pre-requisite for the activation of downstream events. We used Actin as the loading control for cytoplasmic fraction of cells and HP1α as the loading control for nuclear fraction of the cells. Transfection of cells with miR-24 mimic or inhibitor resulted in no significant change in the distribution of cytoplasmic versus nuclear level of ATF6, so do the miR24^mut^ mimic and inhibitor (Figure S2B).

Another key event of ER-mediated apoptosis is the XBP1 splicing. We designed RT-PCR primers specifically to detect the un-spliced form and spliced form of XBP1. With tunicamycin treatment, we observed an increased accumulation of spliced XBP1 compared to control group, validating the efficiency of our method to detect both forms (Figure- S2C). To test whether miR-24 regulates XBP1 splicing, we determined the expression of both forms of XBP1 in cells with miR-24 overexpression or knocking-down. We detected no difference in either spliced form or unspliced form of XBP1 when miR-24 expression was altered (Figure S2C).

**Figure 3:**
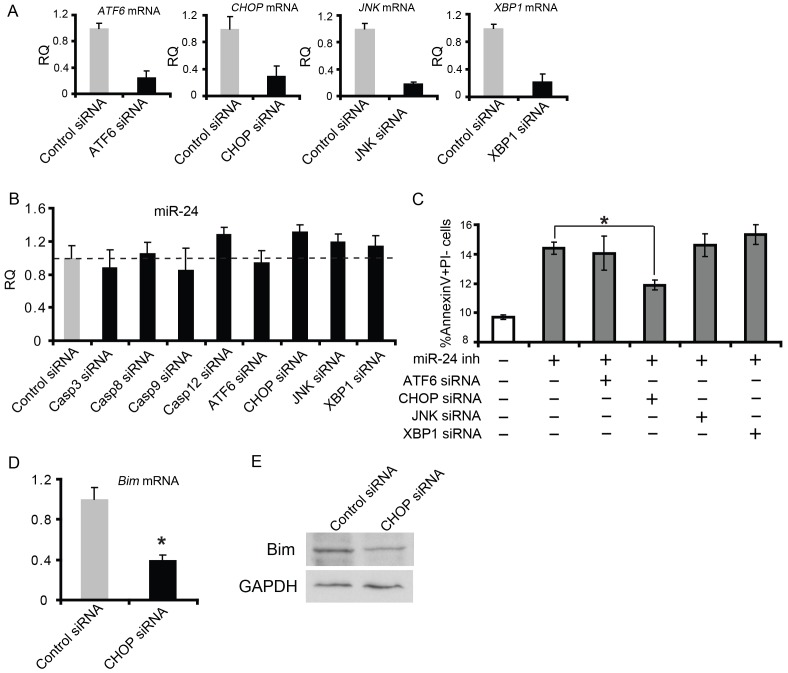
miR-24 inhibits CHOP-induced Bim overexpression in ER mediated apoptosis pathway. (A) Validation of knockdown efficiency using siRNA against *ATF6, CHOP, JNK* and *XBP1* by qPCR. (B) miR-24 expression level was not altered upon knockdown of Caspase 3, Caspase 8, Caspase 9, Caspase 12, ATF6, CHOP, JNK or XBP1. (C) Knockdown of CHOP but not other factors in the ER stress pathway partially rescued increased apoptosis caused by miR-24 inhibition. *, p<0.05. Data were analyzed by unpaired Student's t test. (D) PCR for *Bim* showing decreased *Bim* expression upon CHOP knockdown. *, p<0.05. Data were analyzed by unpaired Student's t test. (E) Western for Bim showing decreased Bim protein level when CHOP siRNA was transfected into primary CMs. GAPDH serves as a loading control.

Apoptosis signals initiated from the ER can also be mediated by increased expression of the transcription factor cytosine-cytosine-adenine-adeninethymine enhancer-binding protein (C/EBP), homologous protein (CHOP), or activation of c-Jun-N-terminal kinase (JNK). As with ATF6 and XBP1, we took similar approaches to determine if miR-24 is involved in these critical events. Manipulation of miR-24 levels in cardiomyocytes did not alter mRNA or protein levels of CHOP or JNK or the phosphrolation of JNK (Figure S2D, E), suggesting CHOP and JNK are not directly regulated by miR-24. We then designed epistasis experiments to test the role of CHOP, JNK, ATF6 and XBP1 in miR-24 mediated apoptosis. First we used siRNA against each gene to down regulate their expression (Fig 3A). Knocking down of ATF6, XBP1, CHOP, JNK and caspases had little effect on miR-24 expression, suggesting that miR-24 does not function downstream of these pathways (Fig 3B). However, we observed that knockdown of CHOP but not any other gene we tested above attenuated the increased apoptosis induced by miR-24 inhibitor (Fig.3C). Since increased Bim protein levels appeared to be a major mediator of apoptosis upon miR-24 inhibition in other cell types [35], [40], we tested whether CHOP could regulate Bim in murine cardiomyocytes. Indeed, CHOP knockdown resulted in a decrease in Bim mRNA and protein levels, suggesting that CHOP normally upregulates Bim as it promotes apoptosis (Fig. 3 D and E). Thus, miR-24 may negatively regulate ER stress-mediated apoptosis in part by preventing the increase in Bim protein associated with CHOP activity. Alternatively, miR-24 could downregulate Bim expression level to counteract the CHOP induced Bim upregulation.

### miR-24 regulates mitochondrial apoptosis pathway by inhibiting Bax translocation from cytosol to mitochondria

**Figure 4:**
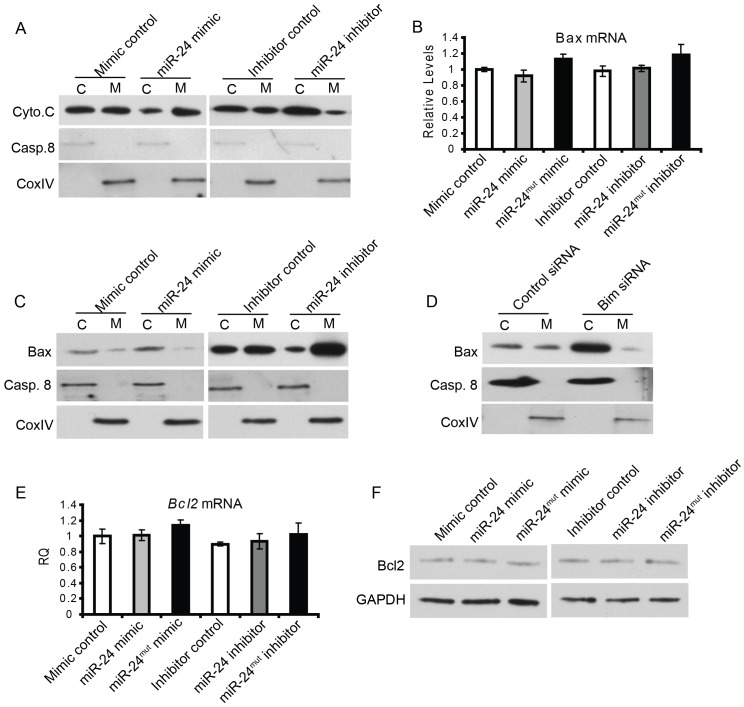
miR-24 regulates mitochondria-mediated apoptosis pathway. (A) Overexpression of miR-24 suppresses cytochrome C release from mitochondria to cytosol. Western blot for cyto.C in cytosol (C) or mitochondria (M) fractions of primary cardiomyocytes transfected with miR-24 mimic, inhibitor, or corresponding controls. Caspase 8 was used as a loading control for cytosol fraction; CoxIV was used as a protein marker for the mitochondrial fraction. (B) qPCR for *Bax* mRNA from primary cardiomyocytes transfected with miR-24 mimic, inhibitor and corresponding controls. (C) Western blot for Bax on cytosol (c) or mitochondrial (m) fractions of primary cardiomyocytes transfected with miR-24 mimic, inhibitor and corresponding controls. (D) Western blot for Bax on cytosol (c) or mitochondrial (m) fractions of primary cardiomyocytes transfected with control or *Bim* siRNAs. (E) qPCR for *Bcl2* mRNA from cells transfected with miR-24 mimic, inhibitor or corresponding controls. (F) Western blot for Bcl2 protein showing miR-24 did not regulate the protein level of Bcl2.

After we determined the role of miR-24 in ER-mediated apoptosis, we next assessed its potential role in mitochondria-mediated apoptosis. The mitochondrial pathway of apoptosis starts with the permeabilization of the mitochondrial outer membrane, which then leads to the release of Cytochrome C from mitochondria to cytosol. Cytochrome c in conjunction with apoptosis protease activating factor (APAF-1) and pro-caspase 9 form an ‘apoptosome’. This complex promotes the activation of caspase 9, which in turn activates effector caspases that collectively orchestrate the execution of apoptosis. To determine whether miR-24 regulates the mitochondrial apoptosis pathway, we first assessed the Cytochrome C release from mitochondria in primary Campothecin-treated cardiomyocytes transfected with miR-24 mimic, inhibitor, or controls. Strikingly, we found reduced translocation of Cytochrome C from the mitochondria to the cytosol, the key event in mitochondria-mediated apoptosis, when miR-24 was overexpressed in cardiomyocytes (Fig. 4A left panel). Conversely, inhibition of miR-24 resulted in increased accumulation of Cytochrome C in the cytosol (Fig. 4A right panel). Cytosolic or mitochondrial fractions were marked by Caspase 8 or the cytochrome oxidase IV subunit.

Next, we examined how upstream factors in this pathway were affected by miR-24. The permeabilization of the mitochondrial outer membrane, the initial step of mitochondrial-induced apoptosis, can be regulated by a variety of Bcl2 family proteins[10]. Among them, the translocation of various Bcl2 family proteins from the cytosol to the mitochondria (or vice versa) is a key event that results in their pro- or anti-apoptotic effects. We therefore examined the expression levels and cellular localization of several key Bcl2 family members including Bcl2, Bax, Bak1 and Bclx in the cardiomyocytes with loss or gain of function of miR-24. We first examined if manipulation of miR-24 would affect the transcription and translation of Bax. We found that neither overexpression nor inhibition of miR-24 resulted in changes in mRNA level of Bax (Fig. 4B). In order to test whether miR-24 modulates Bax translocation in the mitochondria-mediated apoptosis pathway, we separated the cytosolic and mitochondrial fraction of the primary cardiomyocytes and determined the protein levels in the corresponding fraction with altered expression of miR-24. We used CoxIV and Caspase 8 as the positive controls to demonstrate the successful fractionation of cytosol and mitochondria respectively. We found that there appeared to be less Bax in the mitochondrial fraction upon miR-24 expression in primary cardiomyocytes. In contrast, there was an increase in mitochondrial Bax upon miR-24 inhibition (Fig. 4C). Knocking-down of Bim resulted in an increase in the amount of cytosolic Bax compared to mitochondrial Bax (Fig. 4D), suggesting that miR-24 regulates Bax translocation in part through Bim. Interestingly, the increase in cytosolic accumulation of Bax is more pronounced with knocking-down of Bim compared to that of miR-24 mimic treatment. This is consistent with our previous “over-rescue” observation that knocking down of Bim attenuated increased apoptosis caused by miR-24 inhibition to a level that is even lower than the basal level with control transfection [35], suggesting Bim has its independent role in regulating intrinsic apoptosis that is not controlled by miR-24. In contrast, manipulation of miR-24 resulted in limited changes in mRNA, protein expression levels and cellular localization of other Bcl-2 family members such as Bcl-2, indicating that Bcl-2 does not function in miR-24 mediated apoptosis (Fig4 E, F and data not shown). Taken together, these findings reveal a function of miR-24 in repressing the mitochondrial apoptosis pathway by inhibiting Bax translocation to the mitochondria.

## Discussion

Here, we carefully examined the molecular/genetic pathways by which miR-24 regulates programmed cell death in primary cardiomycoytes. We showed that miR-24 modulated intrinsic apoptosis by interacting with CHOP-mediated ER pathways and by inhibiting cytochrome C release, and Bax translocation from cytosol to mitochondria. miR-24 is up-regulated upon cardiac stress in animal models and in humans [41] and may be mediating a compensatory response under cardiac stress. Interestingly, overexpression of miR-24 in primary cardiomyocytes was able to induce hypertrophic growth [41]. Our findings suggest that miR-24 regulation of intrinsic apoptosis pathways might contribute to its ability to modulate apoptosis and possibly hypertrophic growth.

In this study, we demonstrated that Bim and its interacting partners, such as CHOP and Bax, are the major downstream mediators of miR-24 in inhibiting cardiomyocyte apoptosis. However, since a number of pro- or anti-apoptotic genes appeared not to be affected by miR-24 overexpression or inhibition (Figure S2, Fig.4, and data not shown), it is likely that miR-24 represses cell death in diseased hearts through a distinct mechanism that is not part of the canonical apoptosis pathways. In addition, it is still unclear how miR-24 and CHOP exert opposite effects on Bim protein level, and how miR-24 regulates the release of Cytochrome C and the translocation of Bax from cytosol to mitochondria. We have not identified direct binding sites of miR-24 in the UTRs of these genes, thus additional targets of miR-24 might be involved in mediating its effects on Cytochrome C and Bax protein translocation that awaits further characterization.

In addition to its role in regulating apoptosis, miR-24 also functions in the excitation-contraction (E-C) coupling process by targeting the structural protein Junctophilin-2 expression in the cardiomyocytes. Overexpression of miR-24 in the rat primary cardiomyocytes resulted in defective E-C coupling. In a separate study, inhibition of miR-24 was found to be protective on the structural and functional integrity of the ion channel signaling in the hypertrophic aortic-constricted hearts. These studies, in addition to ours, further confirm the endogenous expression of miR-24 in cardiomyocyte. In the heart, miR-24 is also expressed in endothelial cells and cardiac fibroblasts. During myocardial infarction, miR-24 regulates both vascularity and cardiac fibrosis. Thus, this combinatorial effect of miR-24 in different cardiac cell types of the infarcted heart could contribute to the significant cardiac functional improvement upon introduction of miR-24 by either in vivo transfection or virus transduction. Given the nature of microRNAs, it is not surprising that one microRNA can play multiple roles within one organ. However, how one function of miR-24 is executed in a fine manner without interfering with its other function is intriguing.

It is very interesting to note that the role of miR-24 in regulating apoptosis is context-dependent. In certain cell types, such as cardiomyocyte, neural retina and hematopoietic cells, miR-24 is believed to repress apoptosis by inhibiting various pro-apoptotic proteins. While in other cell types including endothelial cells, miR-24 has been shown to target anti-apoptotic genes to promote cell death. Therefore, when it comes to target miR-24 for therapeutic interventions, the cell type specific context has to be considered.

Myocardial ischemia/infarction is a severe stress condition in the heart that can cause significant loss of cardiomyocytes. However, the mechanism that leads to cardiomyocyte apoptotic cell death is only partly defined. Our findings that miR-24 modulates intrinsic apoptosis in murine primary cardiomyocytes might open a new window for gaining additional insights into the role of miRNAs in regulating stress-related heart diseases. In addition, manipulating the expression level of miR-24 and/or its downstream effectors in injured/failing heart may lead to intervention of novel therapeutic strategies.

## Supporting Information

Figure S1 **Caspase 12 is not a direct target of miR-24.** Relative luciferase activity (RLA) in primary cardiomyoyctes expressing the luciferase reporter with Caspase 12 3′UTR and miR-24 mimic, inhibitor, and corresponding controls. Controls are set up as 1. The experiment was repeated three times with biological triplicates (n = 3). Bar graphs show mean±SEM.
(TIF)

Figure S2 **miR-24 does not regulate ATF6, XBP1, CHOP and JNK in ER-mediated apoptosis pathway.** (A) Overexpression of miR-24 mimic, inhibitor and corresponding controls has minimal effect on the mRNA level of ATF6. (B) Western blot for ATF6 from both cytosolic (c) and nuclear (n) fractions of primary cardiomyocytes transfected with miR-24 mimic, inhibitor, and corresponding controls. Actin was used as a loading control for cytosol fraction; HP1α was used as a protein marker for nucleus fraction. (C) RT-PCR showing unchanged proportion of unspliced and spliced forms of XBP1 upon manipulation of miR-24 levels. Samples treated with tunicamycin (Tun) served as positive controls to show effective splicing of XBP1. GAPDH serves as a loading control. (D) mRNA levels of CHOP (upper panel) and JNK (lower panel) were not affected by introduction of miR-24 mimic, inhibitor and corresponding controls. (E) miR-24 does not regulate CHOP protein expression and JNK phosphorylation.
(TIF)
