# Supplementary figures and images for: miR-24 Regulates Intrinsic Apoptosis Pathway in Mouse Cardiomyocytes
Source: PLoS One. 2014 Jan 15;9(1):e85389. doi: 10.1371/journal.pone.0085389 (PMC3893205; doi:10.1371/journal.pone.0085389)

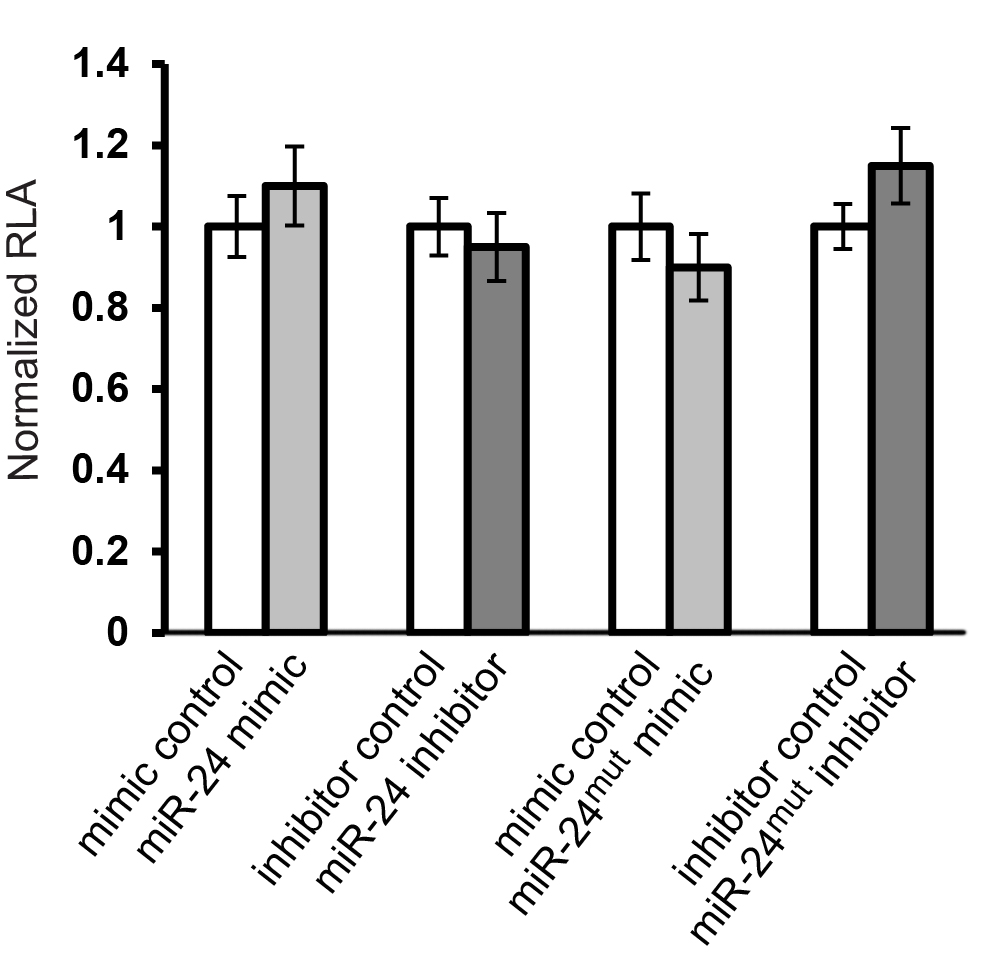

Supplement: Figure S1 — Caspase 12 is not a direct target of miR-24. Relative luciferase activity (RLA) in primary cardiomyoyctes expressing the luciferase reporter with Caspase 12 3′UTR and miR-24 mimic, inhibitor, and corresponding controls. Controls are set up as 1. The experiment was repeated three times with biological triplicates (n = 3). Bar graphs show mean±SEM. (TIF) [file pone.0085389.s001.tif]

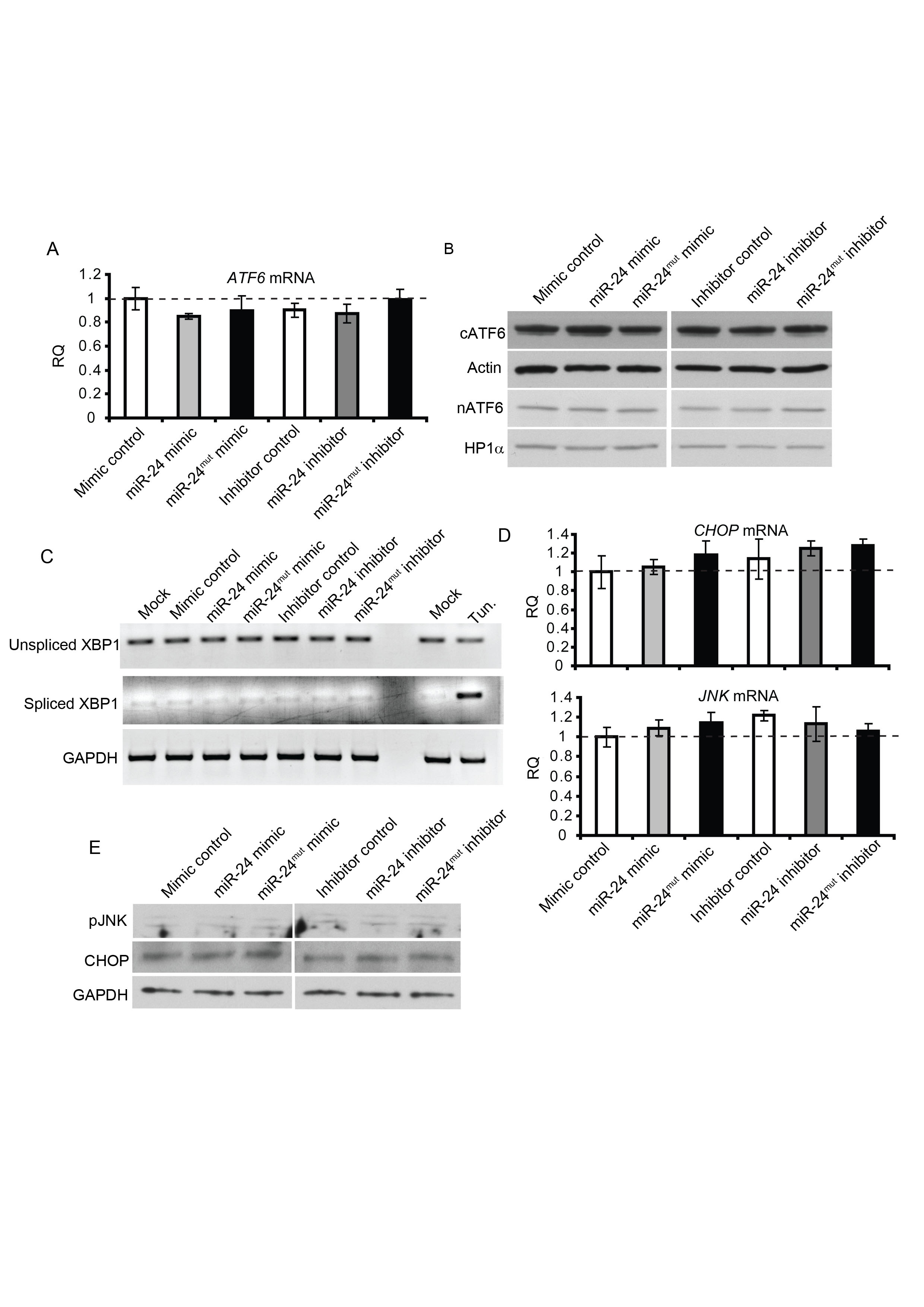

Supplement: Figure S2 — miR-24 does not regulate ATF6, XBP1, CHOP and JNK in ER-mediated apoptosis pathway. (A) Overexpression of miR-24 mimic, inhibitor and corresponding controls has minimal effect on the mRNA level of ATF6. (B) Western blot for ATF6 from both cytosolic (c) and nuclear (n) fractions of primary cardiomyocytes transfected with miR-24 mimic, inhibitor, and corresponding controls. Actin was used as a loading control for cytosol fraction; HP1α was used as a protein marker for nucleus fraction. (C) RT-PCR showing unchanged proportion of unspliced and spliced forms of XBP1 upon manipulation of miR-24 levels. Samples treated with tunicamycin (Tun) served as positive controls to show effective splicing of XBP1. GAPDH serves as a loading control. (D) mRNA levels of CHOP (upper panel) and JNK (lower panel) were not affected by introduction of miR-24 mimic, inhibitor and corresponding controls. (E) miR-24 does not regulate CHOP protein expression and JNK phosphorylation. (TIF) [file pone.0085389.s002.tif]
